# Analyzing Changes in Maize Leaves Orientation due to GxExM Using an Automatic Method from RGB Images

**DOI:** 10.34133/plantphenomics.0046

**Published:** 2023-05-09

**Authors:** Mario Serouart, Raul Lopez-Lozano, Gaëtan Daubige, Maëva Baumont, Brigitte Escale, Benoit De Solan, Frédéric Baret

**Affiliations:** ^1^ Arvalis, Institut du végétal, 228, route de l’aérodrome - CS 40509, 84914 Avignon Cedex 9, France.; ^2^ INRAE, Avignon Université, UMR EMMAH, UMT CAPTE, 228, route de l’aérodrome - CS 40509, 84914 Avignon Cedex 9, France.; ^3^ Arvalis, Ecophysiology, 21 Chemin de Pau, 64121 Montardon, France.

## Abstract

The sowing pattern has an important impact on light interception efficiency in maize by determining the spatial distribution of leaves within the canopy. Leaves orientation is an important architectural trait determining maize canopies light interception. Previous studies have indicated how maize genotypes may adapt leaves orientation to avoid mutual shading with neighboring plants as a plastic response to intraspecific competition. The goal of the present study is 2-fold: firstly, to propose and validate an automatic algorithm (Automatic Leaf Azimuth Estimation from Midrib detection [ALAEM]) based on leaves midrib detection in vertical red green blue (RGB) images to describe leaves orientation at the canopy level; and secondly, to describe genotypic and environmental differences in leaves orientation in a panel of 5 maize hybrids sowing at 2 densities (6 and 12 plants.m^−2^) and 2 row spacing (0.4 and 0.8 m) over 2 different sites in southern France. The ALAEM algorithm was validated against in situ annotations of leaves orientation, showing a satisfactory agreement (root mean square [RMSE] error = 0.1, *R*^2^ = 0.35) in the proportion of leaves oriented perpendicular to rows direction across sowing patterns, genotypes, and sites. The results from ALAEM permitted to identify significant differences in leaves orientation associated to leaves intraspecific competition. In both experiments, a progressive increase in the proportion of leaves oriented perpendicular to the row is observed when the rectangularity of the sowing pattern increases from 1 (6 plants.m^−2^, 0.4 m row spacing) towards 8 (12 plants.m^−2^, 0.8 m row spacing). Significant differences among the 5 cultivars were found, with 2 hybrids exhibiting, systematically, a more plastic behavior with a significantly higher proportion of leaves oriented perpendicularly to avoid overlapping with neighbor plants at high rectangularity. Differences in leaves orientation were also found between experiments in a squared sowing pattern (6 plants.m^−2^, 0.4 m row spacing), indicating a possible contribution of illumination conditions inducing a preferential orientation toward east-west direction when intraspecific competition is low.

## Introduction

Maize (*Zea mays L.*) is currently the most important cereal grown globally, with a production of 1.2 billion tons per year [1]. The positive trend observed on maize productivity during the last decades results from the combination of genetic, agronomic, and climatic factors [2]. The selection of maize cultivars with increased density tolerance was instrumental [3,4]. This was confirmed by independent studies showing the importance of genotype when increasing the plant density to reach high yields [5–7].

In environmental conditions where water and nitrogen are not limiting, the relationship between plant density and yield is largely determined by the ability of the plant to deal with intraspecific competition while maximizing light interception. Indeed, maize plants have the capacity of adapting their architecture when increasing plant density or changing plant distribution patterns [8]. The architectural plasticity of maize cultivars to plant density and distribution has been documented in several studies, including changes in leaves inclination and curvature [9] or leaf lamina dimensions and internode heights [10]. Architectural plasticity is therefore an essential trait for breeders to issue improved maize cultivars capable of maximizing yields under high density conditions. Recently, Perez et al. [11] highlighted the importance of architectural traits related with the vertical distribution of leaf area in the selection of modern maize cultivars adapted to high density. For that purpose, it is necessary to identify the genotype-to-phenotype links that are responsible for such plasticity [12].

One of the most interesting plasticity mechanisms observed in maize when facing intraspecific competition is leaf reorientation. Changes in leaves azimuth when increasing plant density has been already documented by some previous studies [8,13], showing that, under highly rectangular distribution patterns (when distance between rows is much higher than distance between plants in the same row), maize plants can turn leaves through directions perpendicular to the row. This would permit to optimize light interception by decreasing mutual shading [14,15]. The study [16] has shown that leaves reorientation in maize is a phytochrome-mediated response to a reduction in the ratio between red and far-red incident radiation (R:FR) in the stem caused by the presence of neighbor plants (see also [17]). In [16], the authors verified this hypothesis on 2 different maize genotypes: one with the ability to reorient its leaves when R:FR decreased and another one insensitive to R:FR resulting in no significant changes of leaves azimuth when rectangularity increases.

The existing works on maize architectural traits and, particularly, on leaves reorientation are limited to 1 or 2 genotypes per study [8,16], which makes difficult to understand the Genotype x Environment x Management interactions behind them. Actually, in situ manual measurements of maize architectural traits, such as leaves orientation, are highly time-consuming, and this has probably limited experimental studies to a small number of genotypes and/or treatments. The recent development of phenotyping systems and interpretation methods [18–20] allows now collecting high-throughput observations of architectural traits. Several studies demonstrated the pertinence of the information provided by high-spatial resolution RGB (red green blue) cameras in ground sensors or onboard unmanned air vehicles to retrieve specific traits including the plant density at emergence [21,22], the number of leaves per plant for juvenile stages [23], or the monitoring leaf rolling under water stress conditions [24]. More recently, other studies have successfully applied segmentation methods to LiDAR 3-dimensional point clouds to estimate individual leaf area and insertion angle of maize plants cultivated in pots [25,26].

To our knowledge, there are no existing works who have tried to develop indirect, automatic methods to describe leaves orientation of maize genotypes under field conditions. Previous studies like [11] have successfully applied automatic methods based on 3D reconstruction to describe the architecture of maize plants grown in pots in greenhouse experiments. The development of automatic methods to track changes in leaves orientation in actual canopies under field conditions remains a challenge. In this context, the objective of the present study is 2-fold. First, this paper proposes an automatic algorithm (Automatic Leaf Azimuth Estimation from Midrib detection [ALAEM]) based on leaves midrib detection in vertical RGB images to describe the distribution of maize leaves orientation at the canopy level in field conditions and validates the algorithm against manual ground measurements. Second, the paper presents the results retrieved when using the algorithm to describe genotypic and environmental differences in leaves orientations in a panel of 5 maize cultivars sowing at 2 densities (6 and 12 plants.m^−2^) and 2 row spacing (0.4 and 0.8 m) over 2 different experimental sites in southern France. Emphasis is put in analyzing the plasticity of the 5 cultivars to reorient their leaves when increasing the rectangularity of plant distribution. The advantages and limitations of ALAEM to describe leaf orientation in operational conditions (i.e., in phenotyping experiments) against traditional methods based on in situ measurements or canopy transmittance are also discussed.

## Materials and Methods

### Experimental setup

Two field experiments were conducted, respectively in 2021 at the INRAE Avignon experimental site (43°54′N, 4°52′W, France) and in 2022 at the Montardon station of the Arvalis Institut (43°22′N, 0°20′W, France). In both field experiments, a panel of 5 commercial hybrids was grown: DKC4814, DKC4974, LG 30444, KWS INTELIGENS, and URBANIX. These 5 hybrids belong to the same precocity group (confirmed by phyllochron verification) while expected to express a priori different architectural characteristics on what regards canopy height and leaf inclination.

**Fig. 1.**
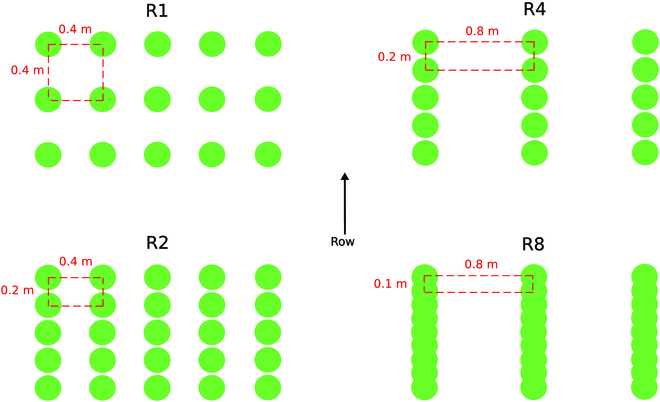
Illustration of the 4 rectangularity levels investigated.

Maize was sown on 2021 May 17 in Avignon and 2022 June 1 in Montardon. In both experiments, the 5 maize hybrids were sown at 4 distribution patterns, resulting from the combination of 2 plant densities (6 and 12 plants.m^−2^) and 2 row spacing (0.4 and 0.8 m). These 4 patterns constitute a gradient in rectangularity (*R*, the ratio between row spacing and plant spacing within the row) from 1 (6 plants.m^−2^ at 0.4 m row spacing) to 8 (12 plants.m^−2^ at 0.8-m row spacing); see Fig. 1. *R* is a variable commonly used to describe sowing patterns [27,28]. Both experiments were conducted under nonlimiting water and nitrogen conditions.

**Fig. 2.**
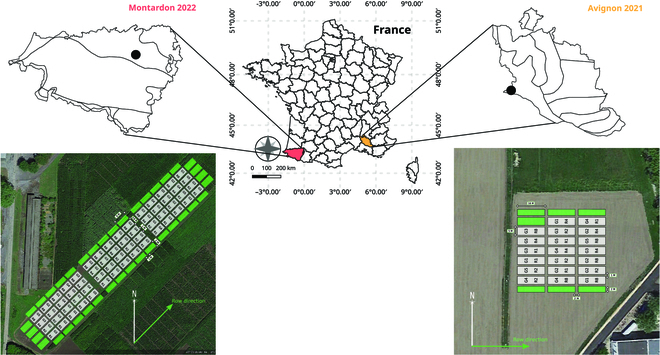
Experimental design in Avignon and Montardon sites with 5 maize hybrids (G1: DKC4814; G2: DKC4974; G3: LG 30444; G4: KWS INTELLIGENS; G5: URBANIX) and 4 sowing patterns (R1, R2, R4, and R8).

A total of 20 unique combinations GxR were evaluated on each experiment. In Avignon, the experimental design consisted in 20 microplots distributed randomly (Fig. 2) of 16 × 4 m size, corresponding to 5 rows (when row spacing was 0.8 m), and 10 rows (when row spacing was 0.4 m). Rows were oriented in the direction east-west (E-W). In Montardon, the experimental design consisted in a 3-block design where each GxR combination was replicated, thus resulting in a total of 60 microplots of 6 × 4 m size, corresponding to 4 rows (when row spacing was 0.8 m), and 6 rows (when row spacing was 0.4 m). To facilitate sowing, the microplots with a given *R* were distributed in the same column (see Fig. 2). The rows in Montardon were oriented approximately in the direction northeast-southwest (row azimuth 42.74°). In both sites, buffer plots were sown at each side of the experiment to prevent possible border effects [29].

### Manual measurements of leaves orientation

Manual measurements of the number of visible and ligulated leaves and the relative azimuth of between ligulated leaves and row direction were taken at 3 dates: at appox. 220 °Cd GDD (growing degree days) after sowing, 430 and 650 °Cd. These 3 dates correspond to, respectively, 3 to 4 visible-leaves stage, 8 to 9 visible-leaves stage, and 12 to 13 visible-leaves stage. These measurements were taken on a sample of 10 and 12 plants per microplot for, respectively, Montardon and Avignon experiments. At the first measurement date, the sampled plants were marked with a white plastic collar. There were distributed in 2 segments of 5 consecutive plants in the central rows of the microplot, trying to prevent possible border effects.

The azimuths of the individual leaves relative to the rows direction were visually determined, and an iron compass was used for directions graduations guidance. Measurements were based on leaves proximal projections [8] to account for the possible twist/shift of the distal part of the blade. At each measurement date, only those leaves not measured in the previous dates were considered (normally, the top 4 to 6 leaves). It is important to mention that the measurements were not conducted under windy conditions. For practical purposes, azimuth angles were reported every 10° for Avignon 2021 experiment but every 30° for Montardon 2022. Reporting the relative azimuths every 30° permitted to reduce substantially the resources needed to sample all the 60 microplots in Montardon, the in situ determination of leaves azimuth is highly time-consuming.

### Indirect estimation of leaves orientation from vertical RGB images

#### RGB image acquisition

Vertical RGB images were taken in both field experiments using a portable handheld phenotyping device developed in the frame of the LITERAL project (funded by the French CASDAR program and led by the Arvalis Institut). This device consists of a pole equipped with 2 SONY RX0 II cameras that are fixed on a support mounted at the tip. The support includes a digital inclinometer permitting us verify the zenith angle of the cameras at each acquisition. Each camera has a field of view (FOV) of 70° in the horizontal direction and 50° in the vertical direction and produces RGB images with a size of 4,800 × 3,200 pixels.

**Fig. 3.**
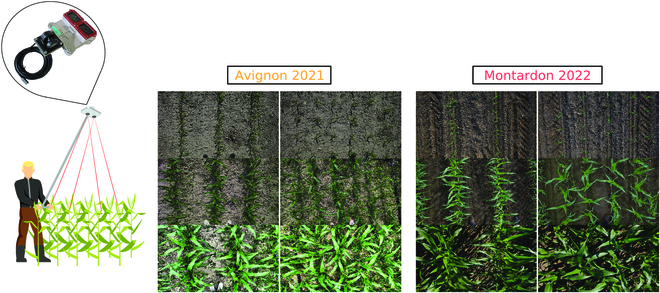
Illustration of image acquisition process and raw image examples.

The images were taken concurrently to the manual measurements in all microplots, again in nonwindy conditions. Twelve image acquisitions per microplot were taken in Avignon, and 6 in Montardon in single longitudinal transects along the rows direction (see Fig. 3). In each transect, the operator was placed in the middle of the central inter-row of the microplot. The length of the pole was adjusted at each date so the position of the camera was, approximately, 2 m, above the top of the canopy (nadir view). Thanks to the digital inclinometer, the camera position was restricted to a maximum of 2° from zenith in every acquisition. This setup provided a spatial resolution of 0.5 mm/pixel and a footprint of 1.4 × 1 m at the top of the canopy and guaranteed an exhaustive sampling of the central rows of each microplot.

#### ALAEM

**Fig. 4.**
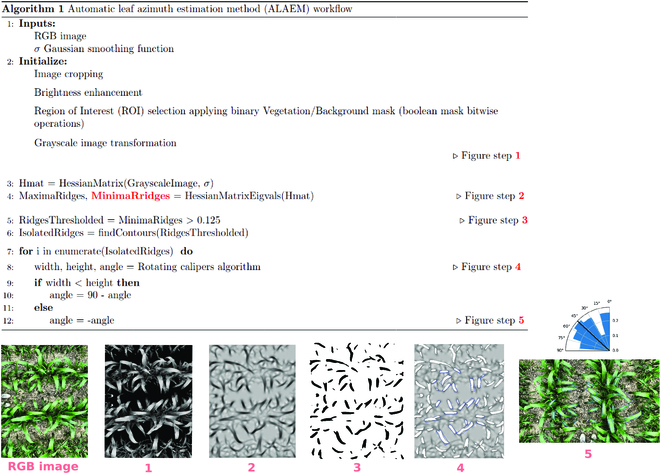
Workflow of the automatic azimuth detection algorithm from vertical RGB images.

The workflow of the ALAEM algorithm is presented in Fig. 4. RGB images were first cropped to extract a ±10° FOV around zenith thus preventing geometric distortions impacting leaves azimuth determination. This FOV corresponds to, approximately, leaves of the 2 central rows. Contrast and brightness enhancements were applied to improve image quality, and an automatic green/nongreen segmentation algorithm [30] to separate the background from healthier leaves.

Leaves midribs are considered as ridges in the images. To detect ridges, first grayscale images (generated by averaging the 3 color channels) are smoothed with a Gaussian filter to remove possible image noise:

$$G\left(x,y,\sigma \right)=\frac{1}{2\pi \sigma^{2}}e^{-\frac{x^{2}+y^{2}}{2\sigma^{2}}}$$

where *σ* is the standard deviation of the Gaussian filter. The parameter *σ* is the only parameter required for the ALAEM method. The adequate value for *σ* changes depending on the size of leaves and, therefore, it should increase with the development stage. Here, *σ* was fixed to 8, 12, and 16 for images taken at 220, 430, and 650 °Cd, respectively.

Then, the Hessian matrix (describing the second-order partial derivative of the smoothed image around each pixel) is constructed as:

$$H_{f\left(x,y\right)}=\left[\begin{array}{cc} \frac{\partial^{2}f}{\partial x^{2}} & \frac{\partial^{2}f}{\partial x\partial y}\\ \frac{\partial^{2}f}{\partial x\partial y} & \frac{\partial^{2}f}{\partial y^{2}} \end{array}\right]=\left[\begin{array}{cc} f_{\mathrm{xx}} & f_{\mathrm{xy}}\\ f_{\mathrm{xy}} & f_{\mathrm{yy}} \end{array}\right]$$

Relative intensity variances on 2 orthogonal directions, defined by eigenvectors, are computed according to the 2 eigenvalues *λ*1 and *λ*2 correspondence from the Hessian. In our case, ridge structures have a small *λ*1, and a nominal threshold value of 0.125 was fixed to classify leaves midrib from other linear features in the image. An automatic Otsu threshold could be theoretically used to identify ridges, but it was not considered here due to nonuniform illumination within images.

A function that highlights the minimum rectangular area enclosing a binary detected ridge was then applied. This function takes as an input the convex hull, i.e., the closed polygonal set of points of the detected ridge. Based on the theorem declared in [31], as the smallest area enclosing rectangle of an object has a side collinear with one of the edges of its convex hull, an iterative loop over each antipodal pairs of vertices/edges of the convex hull is computed. The smallest bounding box gives us the minimum rectangular area. This approach referred to rotating calipers algorithm [32]. Denoising was then applied removing any outliers in rectangular areas shapes due to Hessian matrix artifacts. This method enables averaging over the total length of the midrib and correcting possible twisting effects. Finally, the main rectangle direction relative to the row direction is computed.

The ridge detection step was performed with the help of Scikit-image library in Python. Rotating calipers algorithm with OpenCV library in C++/Python. The full ALAEM code is available at github.com/mserouar/ALAEM along with data samples of each date and GxR conditions with a reproducible example.

### Reliability assessment of ALAEM to describe leaves orientation distribution

As circular data has periodic nature, multimodal distributions analysis can therefore be sensitive and biased depending on a priori assumptions of the model used to best fit the real distribution. As previously mentioned, angle measurements were expressed in [0,90°] range, considering the row direction as the reference (0°). However, the unequal sample size effect between manually measured plants faced to entire plot may lead to reduced statistical power issues. The decision to focus our analyses on the relative proportion/frequencies of leaves oriented perpendicularly to rows instead of distributions was then chosen to validate the ALAEM method. Data extracted from the algorithm distributions are continuous.

The direction of the midribs detected by the ALAEM algorithm were validated against the manual measurements in situ for each Genotype x Site x Rectangularity treatment. The indicator chosen to perform this analysis was *fp*, the fraction of leaves annotated in the field or detected per treatment that were oriented perpendicular to the row [0,1]. To calculate *fp*, a threshold of 60° relative to the rows direction was considered in Montardon. Since in Avignon the number of leaves mesured per treatment was smaller, the threshold for *fp* was enlarged to 45°. The purpose of using *fp* as criteria to validate ALAEM estimations is 2-fold. First, field measurements in the Montardon experiment were taken considering azimuthal sectors of 30°, which makes it unsuitable to compute robustly and accurately a mean azimuth angle relative to the rows direction (i.e., only 3 bins in the [0,90°] interval). Secondly, as the number of leaves measured in situ per date and treatment is relatively low (between 80 and 170) compared to the number of leaves detected by ALAEM (∼10 times higher), a metric based on the frequency of a bin is probably more robust than an absolute average value.

Another reason is that the number of plants and leaves measured in the field in Avignon is smaller compared to Montardon. An increase in the bin size to 45° in order to have a realistic metric to validate ALAEM was necessary in Avignon experiment.

### Statistical data analysis

#### The Additive Main effects and Multiplicative Interactions model

To analyze the GxExM interactions, the choice of a linear model may lead to incorrect interpretation. When such strong preliminary model assumptions about trends are made, differences between terms may not reflect the in situ behaviors. In addition, discussion on whether to choose a mixed model in unbalanced data or not (as our selected genotypes can be regarded as a random sample from a larger population) is still a controversial subject in the scientific community.

For these reasons, we preferred to apply the Additive Main effects and Multiplicative Interactions (AMMI) model [33], which would permit to avoid problems of nonlinearity by establishing principal components analysis on the interaction term by transformation of information in a latent space. Two independent AMMI models were constructed for each site, Montardon and Avignon, according to the expression:

$$Y_{\mathrm{ij}}=\mu +G_{i}+R_{j}+\sum_{k=1}^{K}\lambda_{k}b_{\mathrm{ik}}z_{\mathrm{jk}}+\varepsilon_{\mathrm{ij}}$$

where *Y* describes the response variable i.e., frequencies of perpendicular oriented leaves (>45° from the rows direction) of Genotype *i* in Rectangularity *j*, *μ* the overall mean value, *G_i_* a random Genotypic main effect, *R_j_* a fixed Rectangularity main effect, and the random error term, *ε_ij_*, for a given date and site.

Here, the interaction term is rather explained by K multiplicative terms, formed by the product of *λ_k_*. The eigenvalues *b_ik_* and *z_jk_* are the Genotype and Rectangularity principal component scores (eigenvectors) for axis *k*, respectively.

The choice to construct an individual AMMI model for each site to validate the GxE interaction would be more relevant. Indeed, assuming a single epsilon to represent the effect based on experimental sites as an error term is not appropriate. Experimental conditions are quite different, such as the number of replications and the environment between Avignon and Montardon, making difficult to merge together.

#### Relative distance plasticity index

The relative distance plasticity index (RDPI) is an index that ranks species or cultivars according to their phenotypic plasticity and allows to compare statistically the phenotypic plasticity differences [34] over 2 trials. RDPI permits to quantify plasticity per unit of environmental change. In this study, we calculate RDPI to quantify the plasticity of the 5 maize genotypes studied over rectangularity treatments as follows:

$$\mathrm{RDPI}=\sum ‍\frac{\left|X_{\mathrm{ij}}-X_{i\prime j}\right|}{X_{\mathrm{ij}}+X_{i\prime j}}\times \frac{1}{n}$$

where *i* and *i*′ refer to 2 Rectangularity treatments compared, *j* refers to the Genotype considered, and *X_ij_* is the phenotypic value. In this analysis, *X* refers to the average leaves azimuth relative to the rows direction [0,90°] computed from ALAEM estimations. Finally, *n* is defined as the number of pairwise environments.

#### Kolmogorov–Smirnov

A commonly used statistical test to compare any 2 samples distributions, either empirical or theorical, is the Kolmogorov–Smirnov (KS) test [35]. The KS test is nonparametric and widely used to assess the fit quality of a set of data distributions, based on cumulative distribution functions and the maximum distance between those. It eliminates the arbitrary nature and loss of information associated with bin selection, as they make no assumptions about the binning of the datasets. Rejecting the null hypothesis assumes that there are differences between the 2 distributions tested. Unlike *t* tests, which focus on different means analysis, KS tests determine whether samples are drawn from entirely different distributions, not only single direction. In our case, KS tests will be used on [0,90°] raw azimuth angle range for testing first if observed azimuth distributions are significantly different from a uniform distribution, and in a second time, if leaves orientation distribution for each Genotype x Rectangularity treatment is significantly different between the Montardon and Avignon experiments.

## Results

### Validation of the ALAEM algorithm against leaves orientation distribution from in situ measurements

**Fig. 5.**
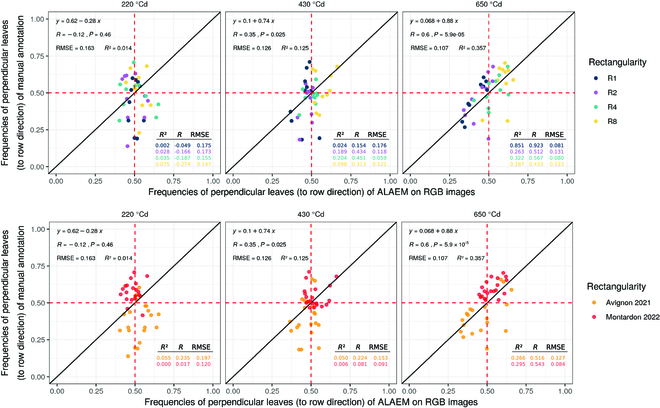
Comparison of the fraction of maize leaves oriented perpendicular to the rows estimated measured in situ and estimated from the ALAEM method at 3 development stages in the Avignon and Montardon experiment. In Avignon, leaves with an azimuth higher 45° relative to rows direction are accounted for to calculate such fraction, whereas in Montardon, only leaves with an azimuth higher than 60° were considered (see Reliability assessment of ALAEM to describe leaves orientation distribution).

Figure 5 shows the comparison between the fraction of leaves oriented perpendicular to the rows direction estimated from the ALAEM algorithm and registered from manual measurements for each GxR combination. The agreement between ALAEM and manual measurements increases progressively with crop development. At 220 °Cd (4 leaves stage), the fraction of perpendicular leaves observed and estimated are practically uncorrelated (*R*^2^ = 0.014, root mean square error [RMSE] = 0.163), and the same applies for the date 430 °Cd (*R*^2^ = 0.125, RMSE = 0.126). It should be noted that on both dates, the total variance of the fraction of perpendicular leaves observed in situ across GxR combinations is higher than the one estimated from the ALAEM method. This is especially true for the Avignon experiment. At 650 °Cd (12 visible leaves), the correlation between the observed and estimated fraction of perpendicular leaves is statistically significant (*P* value << 0.05, *R*^2^ = 0.36), and the ALAEM method describes, overall, most of the observed variability across treatments, genotypes, and sites. The RMSE of the estimated fraction is 10% deviation, which is considered satisfactory.

An important difference between the 3 dates is the number of leaves sampled in both the automatic method and the in situ observation of leaves orientation. As it can be seen in Table 1, the more we move forward along the growing season, the more visible/ligulated leaves are characterized, both manually and with automatic method, as expected since there are more and more leaves to characterized.

**Table 1.** The average number of leaves considered per GxR combination to describe leaves orientation distribution at each measurement date and site by the ALAEM algorithm and manual sampling. The number between parentheses is the average number of leaves per plant, from the top leaf, considered in the in situ measurement followed by cumulative sum between dates).

Method	220 °Cd		430 °Cd		650 °Cd	
	Avignon 2021	Montardon 2022	Avignon 2021	Montardon 2022	Avignon 2021	Montardon 2022
ALAEM	165	620	220	1,109	249	1,406
In situ, manual	45 (3.79 ∣ 3.79)	93 (3.08 ∣ 3.08)	56 (4.69 ∣ 8.48)	126 (4.21 ∣ 7.29)	76 (6.3 ∣ 14.78)	173 (5.77 ∣ 13.06)

Differences between the number of leaves, in automated algorithm on the 2 sites, can be explained by many effects (heterogeneity of the plots due to pest damage, azimuthal configuration masking the leaves of the lower layers, footprint, etc.). On average, this number is 4.5 times higher on Montardon, due to the larger number of images taken per plot and the 3-block design in this site.

### Differences in leaves orientation across genotypes, sowing patterns, and sites using the ALAEM algorithm

**Fig. 6.**
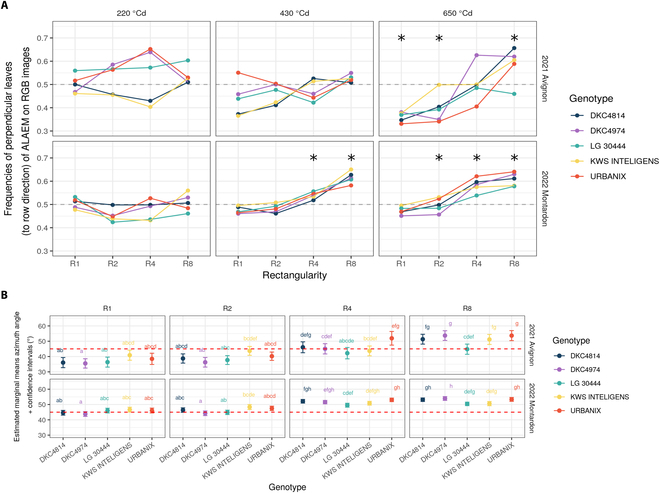
(A) Temporal evolution of the fraction of leaves oriented perpendicular to the rows (>45°) for all the 5 maize hybrid and sowing patterns in the Avignon and Montardon experiments (GxExM per date graph). Asterisks denote dates and rectangularity treatments where at least 3 hybrids present leaves orientation distributions statistically different from a uniform one (KS test). (B) Estimated marginal means of raw azimuth orientation angle for 650 °Cd. Letters indicate significance of differences between the average leaf azimuth angle across hybrids and rectangularity treatments resulting from ANOVA.

Leaf orientation distributions estimated from ALAEM vary depending on genotypes and rectangularity patterns in the 2 experimental sites. These differences become more important as development stage increases. Figure 6A depicts the change of fraction of leaves with azimuth >45° relative to the row directions from 220 to 650 °Cd. For visibility purposes, significance (in black) was plotted only if at least 3 genotypes are different from uniform distribution.

In most cases, a preferential orientation (significative KS test from uniform distribution) of maize leaves can be clearly observed at 650 °Cd (11 to 12 ligulated leaves; see Fig. 6B with raw estimated orientation angles) in both sites Montardon and Avignon. Additionally, also at 430°Cd (8-leaves stage), the observed distribution of leaves azimuth differed significantly from a uniform one at treatments R4 and R8 in Montardon. In most treatments and genotypes, the fraction of leaves oriented perpendicular to the rows is close to 0.5 at 220 °Cd (4-leaves stage), which indicates a homogeneous leaves orientation at early development. The transition from the initial homogeneous distribution at 220 °Cd to the final distribution at 650 °Cd is, according to the observations, progressive.

**Fig. 7.**
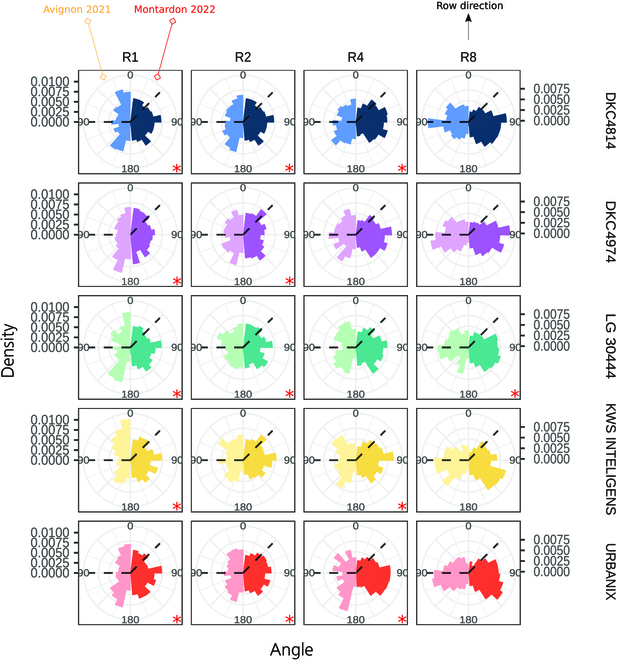
Distribution of leaves azimuth relative to rows direction from the ALAEM algorithm at 650 GDD after sowing (12 visible leaves) for each hybrid, rectangularity, and site. In the polar plots of each site, the N-S directions are indicated with a dashed line.

Figure 7 shows the distribution of leaves azimuth relative to rows direction of the 5 maize hybrids studied at 650 °Cd (12 leaves) depending on rectangularity and site. The angular histograms indicate the influence of rectangularity sowing density pattern in leaves orientation, with a systematic preferential orientation of leaves in direction perpendicular to the rows as rectangularity increases. This preferential orientation in the high-rectangularity treatments is observed at both experimental sites. Interestingly, significant differences in leaves distribution between the sites are found as well for the low-rectangularity treatments (red asterisk in the polar plots of Fig. 7). In the squared pattern (R1), all hybrids in the Avignon site exhibit a strong preference to orient their leaves around the E-W direction (corresponding to the rows direction), whereas no predominant orientation is observed at the Montardon site.

Results on the azimuths relative to the rows observed by ALAEM at 650 °Cd indicate appreciable differences in the mean angle across genotypes for the high-rectangularity treatments. In the treatments R4 and R8, the average leaves azimuth of the DKC4814, DKC4974, and URBANIX hybrids is systematically higher compared to the other 2 (Fig. 6B), indicating a more marked preference of thse 3 hybrids to orientate their leaves perpendicular to the rows. Such differences are statistically significant in the Avignon site only in the R8 treatment, where DKC4974 and URBANIX present a different mean azimuth compared to LG 30444. The KWS INTELLIGENS hybrid exhibits an intermediate behavior between LG 30444, on one side, and DKC4814, DKC4974, and URBANIX, on the other side. These differences among genotypes in the R8 treatment are appreciable as well in the Montardon site (higher mean relative azimuth of URBANIX and DKC4974) but are not statistically significant but showing however a similar trend. This may indicate less need for reorientation due to good ability on other functional traits (inclination, surface, height, etc.), which does not penalize the plant at the end.

**Fig. 8.**
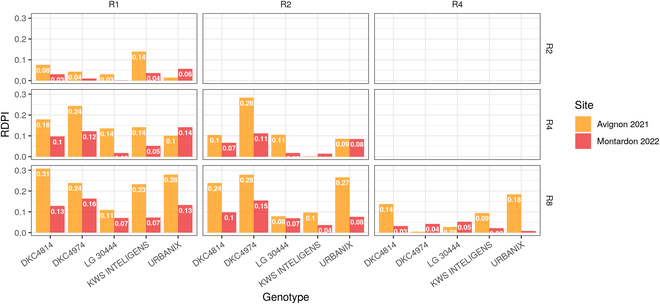
RDPIs pairwise comparison between environments for all genotypes at 650 °Cd.

Figure 8 describes pairwise RDPI across rectangularity treatments for both Montardon and Avignon sites independently. The RDPI computed between R1 and R8 treatments, and between R2 and R8 treatments indicates differences in leaves dynamics/reorientation due to rectangularity. RDPI are systematically higher in DKC4814, DKC4974, and URBANIX, compared to LG 30444 and KWS INTELLIGENS that, according to this indicator, exhibit a less plastic behavior. The absolute values of the RDPI are higher in the Avignon site since all hybrids in the R1 treatment present a preferential orientation parallel to the row (Fig. 7), increasing the differences in leaves azimuth between low- and high-rectangularity treatments compared to Montardon.

**Fig. 9.**
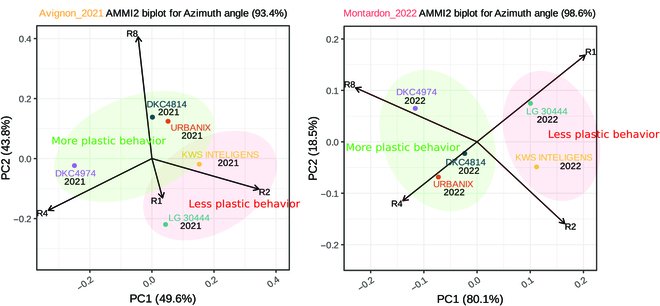
AMMI biplot principal component analysis representation at 650 °Cd.

The results of the AMMI model quantitatively confirm these trends of clusters in genotypes for Montardon site according to their more or less plastic behavior through rectangularity. Table 2 confirms the significance of cited behavior (*F* = 35.6 and a significant *p*-value of 0.048) on principal component 1 (PC1), explaining the largest part of variance (80%). If we focus on Rectangularity biplot positions (Fig. 9), the strength of the interaction is governed by the distance of the environment vectors and genotypes sectors, i.e., genotypes points that are placed in the same direction as the given environments arrows are considered winning genotypes in those environments. Thus, genotypes DKC4814, DKC4974, and URBANIX, for both sites, follow the behavior of Rectangularity R4 and R8, i.e., preferential orientation perpendicular to the row on average and so more pronounced plasticity. Reciprocally for LG 30444 and KWS INTELIGENS on R1 and R2.

**Table 2.** Analysis of variance table for AMMI models for, respectively, Avignon and Montardon. (*) *P* ≤ 0.05, (**) *P* ≤ 0.01, (***) *P* ≤ 0.001.

	Df	Sum sq	Mean sq	*F* value	*P* value (>*F*)	Sum sq	Mean sq	*F* value	P *v*alue (>*F*)
Rectangularity	3	0.16	0.05	14.82	0.00024 (***)	0.06	0.02	35.6	2.965e-06 (***)
Genotype	4	0.02	0.006	1.59	0.23	0.004	0.001	1.8	0.2
Interactions	12	0.04	0.003			0.007	0.0006		
PC1	6	0.02	0.003	2.5	0.3	0.005	0.001	19.7	0.048 (*)
PC2	4	0.019	0.005	3.3	0.24	0.001	0.0003	6.85	0.13
Residuals	2	0.003	0.001			0.000099	0.00005		

The spatial representation of Avignon seems to follow the same trends, i.e., both in the clusters and in the correlation of genotypes on the rectangularity sectors, without however expressing any significance over principal component analyses. This can be explained by the lack of data, in particular by the lack of replicates faced to the 3-block design of Montardon.

## Discussion

### Suitability of the ALAEM algorithm to describe maize leaves orientation in field conditions

The ALAEM algorithm proposed in this study permitted to retrieve realistic distributions of maize leaves azimuth in field conditions. The validation of the algorithm against manual measurements indicated that correlation between the fraction of leaves perpendicular to the rows observed in situ and estimated from ALAEM was only satisfactory at advanced development stages (12 leaves, *R*^2^ = 0.35). This is attributed to the lack of representativeness of manual in situ measurements that increases dramatically the variance in the fraction of perpendicular leaves across treatments and genotypes (see Fig. 5). As the manual measurements are taken in a fixed number of plants per microplot, the total number of leaves annotated per treatment is rather small at early developmental stages (220 and 430 °Cd; see Table 1), which explains the large variance observed across treatments and genotypes in the manual observations on early dates. Retrieving realistic distributions of leaves orientation at the canopy level requires measuring in situ a large number of leaves, but manual annotations of leaves azimuth in the field are highly time-consuming. ALAEM permits circumventing the limitations of manual measurements with a minimum parametrization. Compared to object detection algorithms based on convolutional neural networks, frequently used for organ detection, the detection of maize midribs in ALAEM is unsupervised and does not require any training with manual image annotations. The only parameter that needs to be adjusted is sigma (see ALAEM) to avoid a substantial bias in the detection of midribs. The appropriate values for the sigma parameter are a priori neither site- nor hybrid-dependent. Only large changes in leaves size, e.g., given by the development stage, the distance from the camera to the canopy, or the camera FOV, may require an adjustment of the sigma parameter. The cost-efficiency of ALAEM makes it particularly suitable for large phenotyping experiments, where manual measurements of leaves azimuth over hundreds of microplots may be extremely expensive. Previous studies about maize leaves orientation in field plots [8,14] relied on detailed and exhaustive manual measurements but were focused on 1 maize hybrid and a limited number of sowing patterns. Thanks to the cost-efficiency of ALAEM, in the current study, we could describe with a reasonable degree of realism the distribution of leaves azimuth in 20 GxR treatments at each experimental site, including sampling design and repetitions.

Nevertheless, ALAEM presents some shortcomings. As it is based on vertical RGB images, ALAEM cannot provide azimuths per leaf rank, unlike detailed manual measurements. Moreover, only a fraction of the midribs present in the canopy can be actually identified by ALAEM, due to leaves overlapping. This is consistent with the maual measurements that were conducted in the field—which included only a fraction of the top leaves (see Table 1)—but it is not representative of the whole canopy. Especially in advanced development stages, lower-rank leaves are partially or completely hidden in the RGB images, leading to midrib underdetection. Additionally, the illumination conditions during acquisition may influence the ability of ALAEM to detect all the midribs present in the images. The identification of midribs may be more reliable under diffuse illumination conditions, as compared to direct sunlight. Under direct sunlight conditions, often only a part of the midribs are detected. However, this fraction is enough to determine correctly the leave azimuth. Supervised deep learning approaches for semantic edge detection [23,26] can help to improve the detection of fragmented midribs of those leaves that are partially hidden, but at the cost of generating large datasets of annotated images in order to train networks. Consequently, ALAEM provides a distribution that reflects (especially in dense canopies) mostly the orientation of the upper leaves, rather than a complete distribution in the vertical profile, but this would be similar for any optical instrument in field conditions. Instruments providing information about canopy depth, such as LiDAR [36,37] or stereo RGB imaging [38] can help by associating a depth to every leaf/midrib detected in the top layer of the canopy, if linked with several viewing angles. Then, tracking the dynamics of leaves orientation in the top layer by frequent observations (e.g., every 3 to 5 d) should be permit to achieve a complete distribution per leaf rank. Such information will be extremely heavy to set up and challenging in dense canopies but very useful to monitor and understand the onset of a preferential leaves orientation in field conditions.

### Effect of intraspecific competition and environmental conditions in maize leaves orientation

Thanks to the ALAEM algorithm, the current study permitted to study how intraspecific competition and other environmental conditions determined a preferential orientation of leaves for 5 different maize hybrids.

**Fig. 10.**
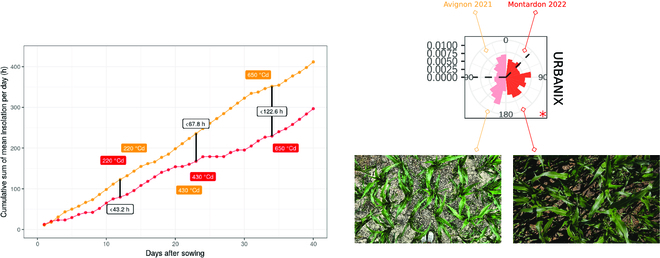
Sunshine hours at different periods between the 4 leaves and the 12-leaves stage in the Avignon and Montardon experimental sites in 2021 and 2022, respectively, and distribution of leaves orientations for the R1 treatment and URBANIX G5 Genotype on both sites, as example.

We observed substantial differences in leaves orientation between the Montardon and Avignon experiments at the squared sowing pattern (R1) for all the 5 hybrids studied. In the study [28], authors also evaluated 4 maize hybrids in a squared sowing pattern, and all of them exhibited a uniform leaves orientation, as in the Montardon experiment. The marked preferential E-W orientation of maize leaves observed in Avignon can be, in principle, explained by differences in illumination/irradiation direct/diffuse conditions against the Montardon site, while they are located at the same latitude and have similar sun track paths. Another hypothesis would be contrasting albedo due to ground reflectance may have induced a different orientation of leaves between Montardon and Avignon. Figure 10 shows the differences in the cumulative sum (over the season) of sunshine hours and a leaves orientation distribution example in both sites for URBANIX cultivar. The number of hours per day with direct sunshine, between 220 and 650 °Cd, in Avignon was substantially higher compared to Montardon. Predominant direct light conditions during summer may have induced maize plants to orient their leaves E-W to maximize light interception in Avignon. Please note that in Avignon, the treatment R2 (0.4-m row spacing, 0.2-m plant spacing) has shown a preferential E-W orientation (Fig. 7). This effect would disappear when conditions are cloudier, as in Montardon. Such effect of direct sunlight in maize leaves orientation at low rectangularity observed in the Avignon experiment has not been previously documented, to our knowledge, and still remains a hypothesis.

At the highest rectangularity (R8), intraspecific competition seems to be the factor determining leaves orientation. The leaf orientation distributions estimated by ALAEM in Montardon and Avignon experiments were similar (see Fig. 7), showing a strong predominance of leaves oriented perpendicular to the row was observed at the 12-leaves stage (650 °Cd after sowing). These results are coherent with the previous findings in [8,16]. However, in the R4 treatment (0.8 row spacing, 0.2-m plant spacing), some moderate differences are found between Avignon and Montardon experiments, possibly induced also by the influence of direct sunlight. Whereas in Montardon, a moderate predominance of leaves orientation perpendicular to the rows was observed for all hybrids, in Avignon, leaves in the R4 treatment tend to be oriented either perpendicularly, either moderately parallel to the rows, i.e., as a bimodal distribution of 45°/135° (DKC4814 or KWS INTELIGENS hybrid; see Fig. 7). In [16], authors demonstrated that plastic maize cultivars reorient their leaves away from neighbors as a reaction to a low local red:far-red ratio (R:FR) in incident light. Consequently, leaves would be oriented perpendicular to the rows in highly rectangular sowing patterns. The shade avoidance mechanism induced by low R:FR ratio seems clearly verified by our study in the R8 treatment at both experimental sites. However, according to our results, in the R4 treatment in Avignon, the shade avoidance mechanism described in [16] could be somehow compensated by the preferential E-W orientation induced by direct sunlight, observed also in R1 and R2. According to this, the reorientation of maize leaves perpendicular to the rows induced by the presence on neighbors would be enhanced in diffuse light conditions, but also under direct light conditions when rows are oriented in the N-S direction. Such hypothesis, however, needs to be further verified. Very few studies have focused on this issue [39].

Our results permitted to identify significant differences among hybrids in their ability to reorient their leaves under high-rectangularity treatments. All the 5 hybrids studied present significant differences in the distribution of leaves orientation between R1 and R8 treatments on both sites (Fig. 6), which indicates some degree of architectural plasticity induced by intraspecific competition. Nevertheless, hybrid LG 30444 presents, systematically, a less plastic behavior (see RDPI in Fig. 8), showing a lower proportion of leaves oriented perpendicular to the rows in the high-rectangularity patterns as compared to other cultivars. By contrast, DKC4814, DKC4974, and URBANIX are those exhibiting the highest plasticity out of the 5 hybrids studied, with higher differences in leaves orientation between low- and high-rectangularity patterns. The study of Maddonni et al. [16] was the first differentiating between rigid and plastic maize cultivars depending on whether cultivars reacted to low R:FR caused by neighbors in their experimental setup. Rather than opposite plastic and rigid behaviors, in our study, we observed a gradient in the ability of cultivars to orient their leaves perpendicular to the rows in the high-rectangularity treatments. Recently, Perez et al. [11] evaluated a panel of 60 maize cultivars grown under controlled conditions. In their study, they found a very low heritability of leaf orientation as compared to traits describing the vertical leaf distribution. However, the sowing pattern was equivalent to the R2 treatment of the present study. Also, greenhouses were used that may affect the radiosity. According to our results (Figs. 6 and 7), at low rectangularity (R1 and R2), the differences among cultivars in leaves orientation are small (in any case, Montardon and Avignon sites), which can explain the low heritability observed in that study.

## Data Availability

The ALAEM algorithm is made public: https://github.com/mserouar/ALAEM.
